# Multi-Elements in Source Water (Drinking and Surface Water) within Five Cities from the Semi-Arid and Arid Region, NW China: Occurrence, Spatial Distribution and Risk Assessment

**DOI:** 10.3390/ijerph14101168

**Published:** 2017-10-02

**Authors:** Ting Wu, Xiaoping Li, Tao Yang, Xuemeng Sun, Howard W. Mielke, Yue Cai, Yuwei Ai, Yanan Zhao, Dongying Liu, Xu Zhang, Xiaoyun Li, Lijun Wang, Hongtao Yu

**Affiliations:** 1 Department of Environmental Science, School of Geography and Tourism, Shaanxi Normal University, Xi’an 710062, China; lanmiao0913@snnu.edu.cn (T.W.); yangfan2288@163.com (T.Y.); sunxuemeng@snnu.edu.cn (X.S.); caiyue@snnu.edu.cn (Y.C.); aiyuwei@snnu.edu.cn (Y.A.); zhaoyanan@snnu.edu.cn (Y.Z.); liu_dongying@yeah.net (D.L.); zhangxu_00@snnu.edu.cn (X.Z.); LeeX_yun@163.com (X.L.); lijun_88@163.com(L.W.); 2 SNNU-JSU Joint Research Centre for Nanoenvironment Science and Health, Shaanxi Normal University, Xi’an 710062, China; hmielke@tulane.edu (H.W.M.); hongtao.yu@morgan.edu (H.Y.); 3 Department of Pharmacology, Environmental Signaling Laboratory, Tulane University, New Orleans, LA 70112, USA; 4 School of Computer, Mathematical and Natural Sciences, Morgan State University, Baltimore, MD 21251, USA

**Keywords:** multi-elements (MEs), drinking water (DW), surface water (SW), health risk

## Abstract

The purpose of this study was to identify the concentration of multi-elements (MEs) in source water (surface and drinking water) and assess their quality for sustainability. A total of 161 water samples including 88 tap drinking waters (DW) and 73 surface waters (SW) were collected from five cities in Xi’an, Yan’an, Xining, Lanzhou, and Urumqi in northwestern China. Eighteen parameters including pH, electrical conductivity (EC), total organic carbon (TOC) total nitrogen (TN), chemical compositions of anions (F^−^, Cl^−^, NO_3_^−^, HCO_3_^−^, SO_4_^2−^), cations (NH_4_^+^, K^+^, Na^+^, Ca^2+^, Mg^2+^), and metals (lead (Pb), chromium (Cr), cadmium (Cd), copper (Cu)) were analyzed in the first time at the five cities . The results showed that pH values and concentrations of Cl^−^, SO_4_^2−^, Na^+^, K^+^, Ca^2+^, Mg^2+^ and Cd, Cr, Cu in DW were within the permissible limits of the Chinese Drinking Water Quality Criteria, whereas the concentrations of other ions (F^−^, NO_3_^−^, NH_4_^+^ and Pb) exceeded their permissible values. In terms of the SW, the concentrations of F^−^, Cl^−^, NO_3_^−^, SO_4_^2−^ were over the third range threshold i.e., water suitable for fishing and swimming of the Surface Water Quality Standards in China. The spatial distributions of most MEs in source water are similar, and there was no clear variation for all ions and metals. The metals in DW may be caused by water pipes, faucets and their fittings. The noncarcinogenic risk of metals in DW for local children are in decreasing order Cr > Cd > Pb > Cu. The carcinogenic risk from Cr exposure was at the acceptable level according to threshold of USEPA. Although the comprehensive index of potential ecological assessment of Cr, Cd, Pb and Cu in SW ranked at low risk level and was in the order of Huang River in Xining > Peaceful Canal in Urumqi > Yan River in Yan’an > Yellow River in Lanzhou, their adverse effects to ecology and human health at a low concentration in local semi-arid and arid areas should not be ignored in the long run.

## 1. Introduction

Multi-elements (MEs) including cations, anions, heavy metals (HMs) and total organic carbon (TOC) and total nitrogen (TN) in drinking water (DW) and surface water (SW) are of major concern because they are persistent and bio-accumulative in nature, and they may pose a potential health risk to residents in urban areas [1]. Water quality and the corresponding health risks have been reported from many parts of the world (i.e., USA, Bangladesh, Pakistan, Turkey, India, China, Europe) [2,3,4,5,6,7,8]. Intensification of industrial, urban development, and agricultural activities have severely degraded the quality of water sources worldwide, in particular, toxic element levels in the aquatic environment have increased [8]. Enrichment of toxic elements in water systems results in the water being unsuitable for drinking, irrigation, fishing, and industrial production. Access to high quality water sources is one of the decisive factors for global, regional, and local development, especially in semi-arid and arid regions. The supplied water quality and suitability for diverse uses are a challenge for most countries. The water resources in China are limited. Due to less precipitation associated with the semi-arid and arid climate, water supply in northwestern China (NW) depends on the surface waters of rivers, so the water resources in NW are not only a valuable natural resource, but also a limiting factor for sustaining the ecological qualities of the environment [9].

Water resources are used in industry and agriculture to develop the national economy, and sustain lives of all human and living organisms. However, the distribution of water resources in China is unbalanced both in space and time. Global weather systems distribute more water in the south and coastal areas and less water in the NW where most of the farmland in China is located. Also, the south and coastal areas receive rainfall during the summer while in NW China’s farmland areas rainfall is sparse during the summer growing season. Moreover, the Chinese per capita water resources were only 2100 cubic meters in 2012, which is a quarter of the world’s per capita value. China was listed by the United Nations as one of the 13 countries facing water shortages. In addition to the NW’s shortage of water quantity, the problem of water quality is worsening too. The situation has received attention by society, especial in the semi-arid and arid regions of NW China. In particular, cations, anions, metals, TOC and TN have frequently increased in the water system recently [10] due to natural processes and anthropogenic activities, such as metallurgy, mining, coal combustion, and metal smelting that release MEs into the surface and drinking water system [11,12,13]. Enrichment of toxic MEs in water systems results in the water being unsuitable for drinking as well as for various industrial and agricultural purposes [14]. It has been reported that ME intake can lead to life-threatening cancers and mental disease [10]. Potential threats to human health and aquatic ecosystems make ME pollution of water systems an ongoing environmental problem [15]. In China, most of the research on MEs contamination has focused on toxic elements in the Yangtze and Yellow rivers [16,17]. Little information on ME contamination, water chemistry and health risk evaluation in NW China is available, although such information is critical for source water management. Therefore, it is important to understand the ME sources, concentration, water chemistry, and health risk levels, as well as water quality, to protect water resources and control water pollution [18].

The surface water in NW China serves as the main source of drinking water for inhabitants living in cities. Surface water, such as the Yellow River, is distributed in Qinghai, Gansu, Ningxia and Shaanxi provinces, respectively, in the semi-arid and arid regions of NW China. Surface water also provides for agricultural irrigation and industrial development. In recent decades, extensive industrial activities, domestic sewage, runoff from agricultural land, and rapid industrial growth have severely deteriorated the water quality in those regions. Such activities have contributed to the increasing ME pollution of the surface rivers.

Measurement of MEs in sources of drinking and surface waters in semi-arid and arid regions of NW China is extremely important for hazards assessment associated with their use. However, due to the large area and small population density, it represents an underdeveloped area in China, so there is little research on their quality analysis. The aim of this study is to evaluate concentration, distribution, sources, and health risk of MEs water quality in drinking and surface waters in five cities from the semi-arid and arid region of NW China. Furthermore, from the measured MEs we also calculate the health hazards of drinking water to inhabitants of cities as well as ecological health of aquatic organisms from MEs in river water. The results are significant and could be used as guidance to water quality and security in the semi-arid and arid region of worldwide.

## 2. Materials and Methods

### 2.1. Study Area

**Figure 1:**
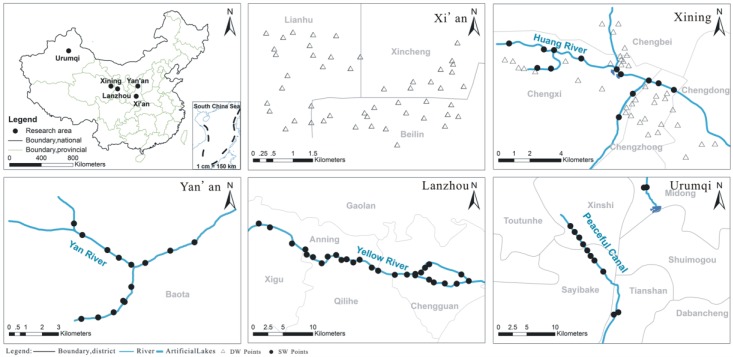
The study area and sampling locations map.

The drinking water and surface rivers samples (*n* = 161) were collected in a climate transition zone corresponding to five areas in Xi’an, Yan’an, Xining, Lanzhou, and Urumqi cities, respectively. A study area map with the sampling sites and locations is shown in Figure 1. The study areas belong to the semi-arid and arid regions of NW China, and the gross water resources are quite limited due to low rainfall, drought, and high rates of evapotranspiration. According to the latest statistics, there are about 20 million people in the 48,618 km^2^ of study area, 82.2% of them use water derived from the Yellow River and its tributaries for drinking, irrigation, industrial development, and the ecosystem service. The source of water for Urumqi is meltwater from mountain glaciers and thus it is more stable and reliable.

### 2.2. Sampling and Analytical Procedure

#### 2.2.1. Sampling

A total number of 88 DW from water faucets and 73 SW from urban rivers were collected. Residential DW was collected from the water faucet after pre-flushing for 5–10 min to remove any stagnant water. Urban SW was collected at a depth of approximate 10 cm from upstream to downstream of the city river (Figure 1) to ensure that the sampled water was representative in the summer of 2015. Some SW samples were taken from the sites near the sewage drain exit. There was no rainfall during the sampling periods. All the water samples were collected in pre-cleaned capped polyethylene bottles (50 mL × 2 for each sample) and the water samples for metal analysis were collected in acid washed polyethylene bottles and preserved by adding nitric acid at the site (pH < 2). The pH and electrical conductivity (EC) were measured in situ. Then the samples were delivered under refrigerated conditions (4 °C in cooling boxes) within 48 h to the laboratory. Once they arrived in the lab, the samples were stored in the dark at 4 °C until they were analyzed. The sampling locations are shown in Figure 1.

#### 2.2.2. Analytical Procedure

The water samples were processed and analyzed for 18 parameters, specifically pH, electrical conductivity (EC), fluoride (F^−^), chloride (Cl^−^), bicarbonate (HCO_3_^−^), nitrate (NO_3_^−^), sulfate (SO_4_^2−^), ammonium (NH_4_^+^), potassium (K^+^), sodium (Na^+^), calcium (Ca^2+^), magnesium (Mg^2+^), cadmium (Cd), chromium (Cr), lead (Pb),copper (Cu), total organic carbon (TOC) and total nitrogen (TN).

All samples were analyzed following the water standard methods published by the national criteria of China (GB/T 6682-2008). The pH and EC were measured at 25 °C using a precision pH meter (PHS 2C) (Nanjing T-Bota Scietech Instruments & Equipment Co., Ltd, Nanjing, China) and EC meter (DDSJ 308A) (Ningbo Biocotek Scientific Instrument Co., Ningbo, China). Fluoride, chloride, nitrate, sulfate, ammonium, potassium, sodium, calcium and magnesium were analyzed by ion chromatography (IC) using an ICS-1500 instrument (Dionex, Sunnyvale, CA, USA). Bicarbonate was analyzed by an auto-potentiometric titrator and total dissolved solid (TDS) was calculated by summing up all major ions. Atomic absorption spectrometry (AAS) used to measure cadmium, copper, chromium and lead by the graphite furnace method was performed using a ZEE nit 700P instrument (Analytic Jena, Jena, Germany). TOC and TN were analyzed by total organic carbon/total nitrogen analysis using an Elementar Vario TOC instrument (Elementar, Langenselbold, Germany). The quality assurance/quality control (QA/QC) was coordinated through careful standardization and procedural blank measurements. The MEs concentration of each sample was within ±~2%. Parameter quantification was determined using a calibration curve of standard solution purchased from National Pharmaceutical Group (Beijing, China).

### 2.3. Heavy Metal Risk Assessment

#### 2.3.1. Drinking Water Exposure and Children Health Risk Assessment

According to the USEPA Handbook on the Exposure Factor [19], there are three pathways to estimate the average daily dose (*ADD*) (mg·kg^−1^·day^−1^) of an element, ingestion, dermal and inhalation respectively. Considering that the main exposure pathways are ingestion and dermal via the drinking water in China, we used the following equations:

(1) $$ADDingest=\frac{C\times IngR\times EF\times ED}{BW\times AT}$$

(2) $$ADDdermal=\frac{C\times SA\times AF\times ABS\times ED\times EF}{BW\times AT}$$

where *C* is the concentration of the element in mg·L^−1^, *IngR* is the ingestion rate in L·day^−1^, *SA* is the surface area of the skin exposure to pollutants in cm^2^, *AF* is the skin adherence factor in mg (cm^2^·h)^−1^, *ABS* is the dermal absorption factor, *EF* is the exposure frequency in day·year^−1^, *ED* is the exposure duration in year, *BW* is the body weight in kg, *AT* is the average time in day.

The calculated Hazard Quotient (*HQ*) indicates the non-cancer risk during a lifetime based on the *ADDs* from each exposure route by a specific reference dose (*RfD*). In general, the overall potential non-cancinogenic effects were posed by the more than one chemical element (e.g., i), so the *HQ* value is summed assuming additive effects and then expressed as a Hazard Index (*HI*) [20]. Besides, if there are multiple pathways, a total exposure Hazard Index (*HIt*) could be used to communicate the non-cancer risks through different pathways. The *HQ*, *HI* and *HIt* are defined as follows [20]:

(3) $$HQ=\frac{ADD}{RfD}$$

(4) $$HI=\sum_{i=1}^{n}HQ$$

(5) $$HIt=\sum_{i-1}^{n}HQ$$

where *RfD* is the estimated maximum permissible risk on humans through daily exposure. There are two thresholds: *RfDing* (mg·kg^−1^·day^−1^) for ingestion and *RfDder* (mg·kg^−1^·day^−1^) for dermal contact. *HQ* ≤ 1 is unlikely to experience adverse health effects, whereas *HQ* > 1 indicates a potential for an adverse effect to occur and a need for further study [21]. Therefore, in the case of *HIt* ≤ 1 the assumption is that no chronic risks are likely to occur and for *HIt* > 1, non-cancer risks are likely to occur, and thus segregating the contaminants and separating *HIt* for the analysis would be appropriate.

For the cancer effect, the Incremental Lifetime Cancer Risk (*ILCR*) is described as the increment probability of an individual developing cancer over a lifetime due to exposure to a potential carcinogen. The *ILCR* is defined as [20]:

(6) ILCR=ADD×SF

where *SF* is cancer slope factor. If there are multiple carcinogenic contaminants, the cancer risk for each carcinogen and exposure route could be added (assuming additive effects) and compared with the acceptable risk. This acceptable level is in the range of 1.0 × 10^−6^ to 1.0 × 10^−4^ [22].

According to the classification group orders defined by the International Agency for Research in Cancer (IARC) on drinking water, Cr, Cd, Cu and Pb were regarded as non-cancer effect elements, while Cr was treated as having potential carcinogen effects [23]. The parameters are listed in Table 1 and Table 2.

#### 2.3.2. Surface Water Ecological Risk Assessment

Ecological risk assessments comprehensively consider the synergy effect, toxic level, concentration and ecological sensitivity of HMs [28,29]. In this study, the HMs in different rivers could be used to estimate the potential risk via following four equations:

(7) $$X_{f}^{i}=\frac{X_{s}^{i}}{X_{r}^{i}}$$

(8) $$X_{d}=\sum_{i=1}^{n}X_{f}^{i}$$

(9) $$E_{r}^{i}=T_{r}^{i}\times X_{f}^{i}$$

(10) $$RI=\sum_{i=1}^{n}E_{r}^{i}$$

where $X_{f}^{i}$ is the contamination factor of single HMs, $X_{r}^{i}$ is the background metal contents using the range I of surface water environmental quality standard in China (mg·L^−1^). $X_{s}^{i}$ is the measured HMs concentration (mg·L^−1^), $X_{d}$ is the comprehensive contamination factor. $T_{r}^{i}$ is the toxic response factor of each elements determined for Cd = 30 > Pb = Cu = 5 > Cr = 2 [30]. $E_{r}^{i}$ is the potential ecological risk index of single HMs, and *RI* was the comprehensive potential ecological risk index. They are classified into four and five levels, respectively, in Table 3 [31].

## 3. Results and Discussion

### 3.1. Hydrochemistry and Natural Variation of MEs

The ME analytical results have been transformed into descriptive statistical parameters. Maximum (Max.), minimum (Min.), median values (Med.), average (AV.) and standard deviation (SD.) for all the analyzed parameters are given in Table 4. The median is the central tendency value and a superior description of the data than the average. As shown, the SD is often larger than the average and this means that the average does not adequately describe the actual data. Please note the units vary between μg/L and mg/L. The dataset is representative of the study area because they have similar geological conditions. All pH, Cl^−^, SO_4_^2−^, Na^+^, K^+^, Ca^2+^, Mg^2+^, Cd, Cr and Cu values of DW are within the permissible limit, whereas F^−^, NO_3_^−^, NH_4_^+^ and Pb exceed the Drinking Water Quality Criteria of China (GB5750-2006). In terms of the SW, F^−^, Cl^−^, NO_3_^−^, SO_4_^2−^ are over the limits according to the third range of the Surface Water Quality Standard of China which is suitable for fishing and swimming in China (GB3838-2002).

#### 3.1.1. The pH and Electrical Conductivity

The pH varies from 7.1 to 8.5 (median 8.0, average 7.9 and SD 0.2) in DW and from 7.0 to 9.0 (median 7.8, average 7.8 and SD 0.37) in SW. Electrical conductivity (EC) is directly related to the concentration of ions dissolved in the water. EC values varies from 114 to 428 μS/cm (median 135.5, average 204.0 and SD 92.1) in DW and from 209 to 3080 μS/cm (median 417.0, average 712.9 and SD 599.1) in SW. Some higher EC values (Figure S2, Supplementary Materials) are from Urumqi water samples which could be caused by evaporation crystallization and precipitation effects.

#### 3.1.2. Anion Chemistry

Fluoride values vary from 0.0 to 3.1 mg/L (median 0.1, average 0.4 and SD 0.6) in DW and from 0.1 to 2.9 mg/L (median 0.7, average 0.8 and SD 0.6) in SW. The fluoride content in many water samples exceeds the guideline values set by China for DW and SW. There is epidemiological evidence that concentrations above this value carry an increasing risk of dental fluorosis and progressively higher concentrations lead to increasing risks of skeletal fluorosis [32]. Water having high chloride is usually taken as a pollution index and considered a tracer for water contamination [7]. Chloride varied from 4.6 to 38.3 mg/L (median 9.4, average 12.0 and SD 6.2) in DW and from 12.5 to 604.8 mg/L (median 81.6, average 125.7 and SD 122.8) in SW. The chloride concentration in surface water thus exceeds the China desirable limit of 250 mg/L at many places. Chloride could be released into rivers through ion exchange processes [33]. HCO_3_^−^ DW concentrations range from 33.2 to 265.3 mg/L (median 58.9, average 88.8 and SD 44.4) and from 36.1 to 880.2 mg/L (median 151.5, average 202.6 and SD 155.8) in SW. Bicarbonate dissolved in surface water is mainly derived from biogenic and mineral sources. In biogenic formation, CO_2_ is released into the soil atmosphere, and thus into waters draining through the soil, both directly from plant roots and by the microbial degradation of soil organic matter [34]. At circumneutral pH, CO_2_ dissolves in water to form bicarbonate. The dissolution of the same carbonate minerals which release Ca^2+^ and Mg^2+^ to solution also yield HCO_3_^−^ [34]. Excessive use of chemical fertilizers is the primary cause of undesirable nitrate levels in water samples [35]. Nitrate concentrations vary from 5.0 to 26.4 mg/L (median 11.2, average 13.0 and SD 4.3) in DW and from 1.4 to 315.0 mg/L (median 17.9, average 39.9 and SD 67.0) in SW. High nitrate concentration in water causes methemoglobinemia or blue baby disease in infants, alimentary canal, respiratory and nervous system disorders [7]. More than half of the samples nitrate exceeds the desirable limit of 10 mg/L. Sulfate is a naturally occurring ion in almost all type of water bodies. Sulfate concentrations vary from 19.0 to 87.0 mg/L (median 26.2, average 36.9 and SD 15.6) in DW and from 30.9 to 2172.9 mg/L (median 148.3, average 274.4 and SD 335.2) in SW. The surface water sulfate concentration exceeds the desirable limit of 250 mg/L in many samples (Figure S2, Supplementary Materials) due to close proximity of the sewage outlets. Sulfate may cause gastro-intestinal irritation at higher concentrations, particularly when Mg^2+^ and Ca^2+^ are also present in drinking water [36].

#### 3.1.3. Cation Chemistry

Ammonium concentrations varied from 0.0 to 1.8 mg/L (median 0.2, average 0.3 and SD 0.4) in DW and from 0.0 to 3.0 mg/L (median 0.0, average 0.1 and SD 0.4) in SW. At many sampling sites, NH_4_^+^ concentrations are above the criteria. Ammonium has a toxic effect on human health only if the intake becomes higher than the capacity to detoxify and ammonium is not of direct importance for health at the concentrations observed in the analyzed waters [37]. Potassium concentration varied from 1.1 to 9.3 mg/L (median 1.9, average 2.3and SD 1.35) in DW and 0.2–97.5 mg/L (median 5.5, average 11.6 and SD 16.2) in SW. The water potassium sources may include rain water, weathering of potash silicate minerals and application of potash fertilizer [7,38]. Sodium varied from 2.1 to 115.7 mg/L (median 4.1, average 8.4 and SD 12.9) in DW and 8.1–1062.3 mg/L (median 78.3, average 140.7 and SD 175.0) in SW. The high sodium value may be related with pollutant discharge. Furthermore, magnesium concentrations varied from 2.7 to 30.0 mg/L (median 3.4, average 6.4 and SD 4.5)in DW and 3.7–64.7 mg/L (median 20.9, average 26.1 and SD 13.0) in SW. China has no specific standard for magnesium, but as a reference, the samples were below the Bureau of India standard of a permissible limit of 100 mg/L [7]. Calcium varied from 16.0 to 73.0 mg/L (median 28.0, average38.6 and SD 15.9) in DW and from 14.6 to 282.3 mg/L (median 41.8, average 60.1 and SD 47.4) in SW. Some river samples exceed the calcium permissible limit of 200 mg/L [7].

#### 3.1.4. The HMs, TOC and TN

HMs may contaminate surface and ground water resulting in deterioration of drinking and irrigation water quality [39].The HMs are considered as severe pollutants owing to their toxicity, persistence and bioaccumulative nature in the environment [40]. Cadmium concentrations varied from 0.0 to 3.6 μg/L (median 0.1, average 0.2 and SD 0.4) in DW and from 0.0 to 0.3 μg/L (median 0.1, average 0.1 and SD 0.1) in SW. Chromium varied from 0.2 to 7.3 μg/L (median 2.5, average 2.5 and SD 1.2) in DW and from 0.4 to 31.7 μg/L (median 3.3, average 4.8 and SD 4.7) in SW. Lead varied between 0.0–14.7 μg/L (median 1.1, average 1.6 and SD 2.1) in DW and 0.0–49.0 μg/L (median 0.5, average 2.7 and SD 8.1) in SW. Copper varied 0.6–161.4 μg/L (median 5.6, average 10.7 and SD 24.0) in DW and 0.2–51.6 μg/L (median 5.1, average 7.2 and SD 7.6) in SW. Although only lead exceeded the permissible limit of 10 μg/L of some samples in DW, the government must pay attention. 

TOC and TN are very important water quality parameters [41]. TOC concentrations varied from 1.6 to 151.6 mg/L (median 3.5, average 5.5 and SD 16.1) in DW and from 0.6 to 47.3 mg/L (Median 3.5, average 5.2 and SD 6.3) in SW. TN varies 4.2–15.8 mg/L (median 6.7, average 7.3 and SD 2.0) in DW and 1.1–100.0 mg/L (median 13.1, average 21.5 and SD 21.9) in SW.

#### 3.1.5. Correlation Analysis

Correlation analysis measures the closeness and the degree of linear association between independent and dependent variables [42]. We used the Spearman correlation coefficient, which is suitable for an independent data distribution, to assess the water quality parameters. An ‘r’ value close to ±1 indicates a close fit to a straight line and an ‘r’ close to 0 indicates a very poor fit to a straight line or little or no correlation. The correlation coefficients (r) among 18 water quality parameters in DW and SW are calculated (Table 5 and Table 6). EC shows positive correlation with most of the elements but pH shows a negative correlation with them in the two source waters, which is in accord with previous studies [7]. F^−^, NH_4_^+^ and Cu are not significantly correlated with any of the parameters studied. Anions (Cl^−^, HCO_3_^−^, NO_3_^−^, SO_4_^2−^) are positively correlated with cations (K^+^, Na^+^, Ca^2+^, Mg^2+^). Na^+^-K^+^ (r^2^ = 0.86), Na^+^-Mg^2+^ (r^2^ = 0.87)and Ca^2+^-Mg^2+^ (r^2^ = 0.83) in DW and Na^+^-K^+^ (r^2^ = 0.74), Na^+^-Mg^2+^ (r^2^ = 0.74) in SW are also the more significant correlation pairs, which shows all of them have the same source [43]. Meharg [44] verified that the metal arsenic was positively correlated in core profiles and co-deposited with organic carbon. Meanwhile, in the Table 5 and Table 6, TOC is positively related with some parameters, including pH, EC, F^−^, HCO_3_^−^, NO_3_^−^, SO_4_^2−^, Na^+^, Mg^2+^, Ca^2+^, Cd, Cr, Pb, and TN has positive correlations with pH, EC, F^−^, HCO_3_^−^, NO_3_^−^, SO_4_^2−^, K^+^, Na^+^, Mg^2+^, Ca^2+^, Cr, Pb, Cu. In general, the correlation coefficients in DW are higher than SW, which is due to the tap water being treated by the waterworks but the natural river quality being uncertain and affected by anthropogenic input.

### 3.2. Multiple-Elements and Regional Distribution

#### 3.2.1. The Spatial Variations and Distributions of MEs

**Figure 2:**
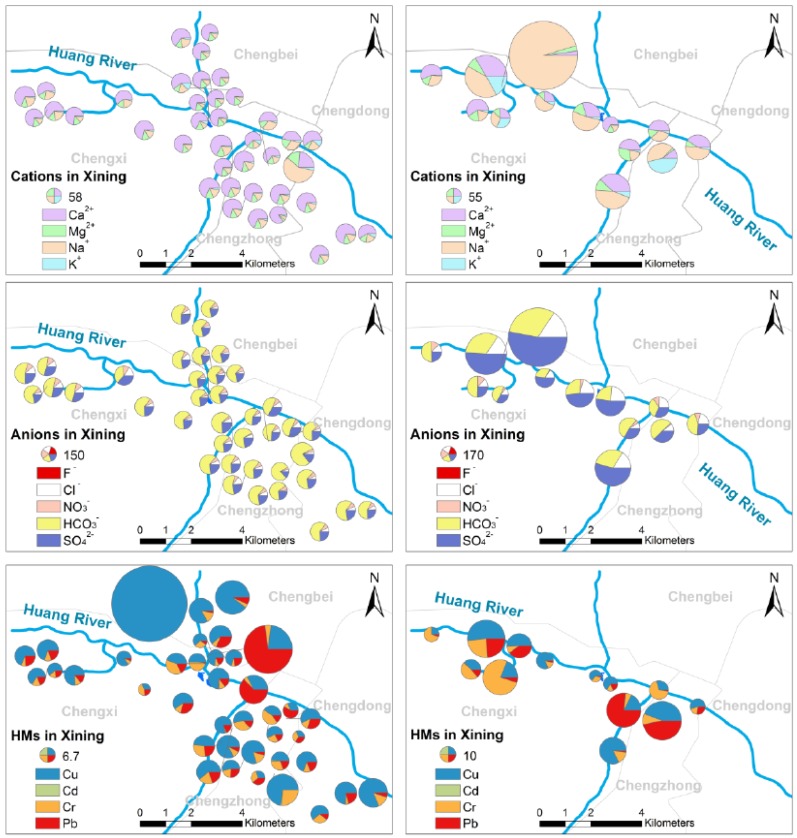
Spatial distributions of cations, anions and HMs of DW and SW in Xining. Notes: The left column as Drinking Water and the right column as Surface Water.

The spatial variations and distributions of MEs in the study area are divided into three parts, cations, anions and HMs, respectively, and the findings are presented in Figure 2 and Figure S1 (Supplementary Materials). After screening the MEs of northwestern China DW and SW, no clear cations and anions variations are found in the waters, but the metals are different. In general, Ca^2+^ and HCO_3_^−^ are the highest cation and anion in DW and the differences in the total ions between each point are not large. However, there are obviously significant differences for ions in the Xining and Lanzhou city rivers. This might be due to the fact the high content sampling point was close to industrial or consumer products outlets. Figure 2 and Figure S1 illustrate that Na^+^ and SO_4_^2−^ were the highest cation and anion in the SW in general, which are influenced by evaporation because of the drought climate in northwest China [7]. The variation among four HMs is obvious. The difference in DW is caused by the water pipes, faucets and their fittings. In terms of the city SW, we find that the proportion of Pb in Huang River is clearly higher than in the other river waters. In addition, the HM results in Xining and Lanzhou city rivers are higher than the other rivers and this is due to the anthropogenic discharges.

#### 3.2.2. The Boxplot of Water Parameters in Different Areas

The occurrences of 18 water parameters are displayed as boxplots in Figure S2 (Supplementary Materials). These boxplots provide evidence to compare the basic statistics of the DW between Xi’an and Xining, the SW between the Yan River, Huang River, Yellow River, and the Peaceful Canal. The following observations can be made:

- The Yellow River seems to have the lowest median values of Cu and TOC, although the differences are not significant.
- The median values of all major elements, except Cd and Cr are significantly higher in Xining DW than in Xi’an DW.
- Yellow River resembles Huang River regarding Na^+^, Cr and TN, and Mg^2+^, Cl^−^, NO_3_^−^, SO_4_^2−^. To this point, we should notice the Huang River is one of branches of Yellow River.
- Xining drinking water resembles the Yellow River regarding Pb, Ca^2+^, Cu and TN, and Mg^2+^, NO_3_^−^, HCO_3_^−^ and TOC also come nearly. In addition, it’s important to note that Huang River is one of the water sources of Xining drinking water.
- Compared to the other datasets, Xi’an DW has a rather uniform content of Na^+^, K^+^, Cl^−^, HCO_3_^−^, SO_4_^2−^ and TN.
- The variation of F^−^ in Xining tap is much larger than others, especially compared with Xi’an DW.

### 3.3. Heavy Metal Risk Assessments

#### 3.3.1. Children Health Risk Assessment of the DW

Amongst three routes of exposure (ingestion, inhalation and dermal absorption), only the ingestion and dermal routes were taken into consideration in this study. Based on the *ADD, RfD* and *SF,* the non-cancer and cancer risks of HMs to the local children were calculated. The *HQs* exhibit a normal distribution after a normality test using the USEPA reference method, the *HI*, *HIt* and *ILCR* of the ingestion and dermal contact pathways are shown for the 5th, median and 95th percentile (Table 7 and Table 8), and the spatial distribution of each *HIt* are observed in Figure S3 (Supplementary Materials). The *HI* and *ILCR* values in all drinking water samples were considered acceptable according to the USEPA guidelines. The *HIt* of HMs decreases in the order of Cr > Cd > Pb > Cu in Xi’an and Xining, indicating that Cr is potentially deleterious to health mainly by the dermal pathway of exposure. It should be especially noticed that the 95% *HIt* value of Cr in Xining was even 0.98. Table 7 and Table 8 indicate that ingestion appeared to be the main pathway for Cu and Pb via water drinking exposure which were in accord with a previous study [45]. As mentioned previously, Cu and Pb in the study area are mainly attributed to faucets and their fittings. Therefore, further research is needed to identify the materials of the faucet and their fittings. Cd and Cr would be the main contributors of human exposure to the water pollutants through dermal exposure. Furthermore, compared with Xi’an and Xining tap water, Xining is higher than Xi’an for 95% *HQ* value, and the main contribution way is both from the content of Cr. The *HI* for HMs from ingestion exposure decreased in the orders of Pb > Cr > Cd > Cu in Xi’an and Pb > Cr > Cu > Cd in Xining. The *HI* for HMs from dermal exposure decreased in the order of Cr > Cd > Cu > Pb in Xi’an and Cr > Cd > Pb > Cu in Xining. And Cd and Cu *HIt* value in Xi’an are higher than Xining, Cr and Pb are contrary to the former. These results indicated that the surrounding environments were not polluted by HMs and people were not exposed to a polluted environment, but we don’t know whether the other HMs and environment media may pose a degree of health risk to people. If they are also considered, the non-carcinogenic risk assessment would be more complicated. However, the sites exceeding the acceptable threshold of *HIt* (*HIt* = 1) are also distinguished and observed in Figure S3 (Supplementary Materials), which implies the toxic metals such as Cr, Cd, Cu and Pb from tap water pipes system would significantly influence the drinking water quality and safety in those locations. The carcinogenic risk from Cr exposure is at the acceptable level (1.0 × 10^−4^), comparatively, the carcinogenic dermal risk levels are 10 times higher than ingestion pathway of exposure. Moreover, when compared with Xi’an and Xining tap water, Xi’an is higher than Xining for 5% *ILCR* value. However, the median and 95% values were very close.

#### 3.3.2. Ecological Assessment of the Surface Water

**Figure 3:**
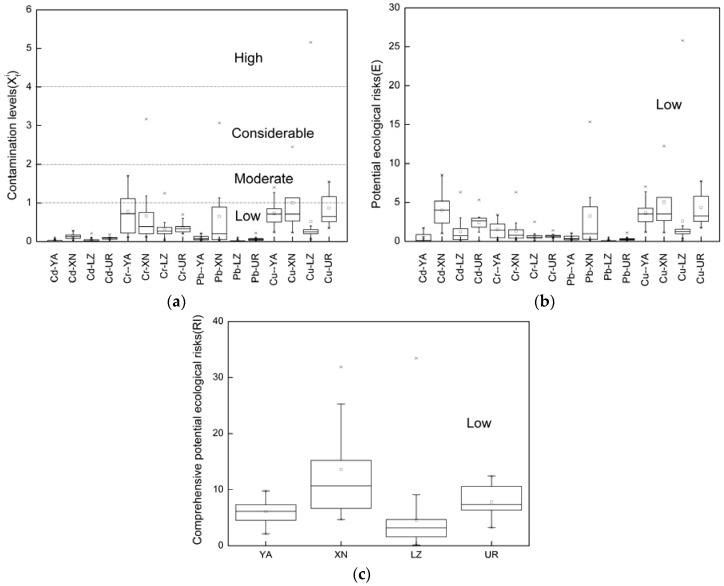
The contamination levels $X_{f}^{i}$ (**a**), potential ecological risk index (E) of HMs (**b**) and the comprehensive potential ecological risks (RI) (**c**) boxplots diagrams in the four city rivers. Note: The horizontal ordinate means: YA means Yan River in Yan’an city; XN means Huang River in Xining city; LZ means Yellow River in Lanzhou city; UR means Peaceful Canal in Urumqi city; Cd-YA stands for heavy metal Cd in Yan River in Yan’an city; Cd-XN for heavy metal Cd in Huang River in Xining city; the heavy metals-abbreviations of city river stands for this metal in the city river in different city, for example, Cd-LZ for heavy metal Cd in Yellow River in Lanzhou city; Cd-UR for Peaceful Canal in Urumqi city. And “*” means the outliers.

According to the Hakanson ecological risk calculation method (Equations (7)–(10) and Table 3), the contamination levels ($X_{f}^{i}$) and potential ecological risk index (*E* and *RI*) were displayed in the Figure 3. It should be noted that an obvious difference exists among the results of the $X_{f}^{i}$, *E* and *RI* analyses. The $X_{f}^{i}$ presents different levels with *E* and *RI* which illustrate that light pollution could not pose a serious ecological risk for river (*X* had four contamination levels from low to high, but *E* and *RI* only had low level), and *RI* mean values calculated for all study city rivers are in the order Xining > Urumqi > Yan’an > Lanzhou. It is observed that the contamination degree of all surface water samples show at the rank of low for mean $X_{f}^{i}$ values, especially the Cd.

Further, the Cr in Yan’an and Lanzhou and the Cu in Yan’an and Urumqi are at low and moderate level, respectively. The Pb, Cr and Cu levels in the Huang River ranked at low, moderate and the phenomenon should draw our attention. In particular, the Cu maximum value in Lanzhou reaches the high level and this may be caused by sewage outfall, while other Cu contamination levels were low.

Although the potential ecological risks all rank at low level, some differences could be observed. For Cr in the Yan River, Cd, Pb, and Cu in the Huang River, the mean potential ecological risk values are higher than for other city rivers. Therefore, Xining city river Huang River indeed has the highest observed potential ecological risk among the four city river. In addition, the maximum of *E* and *RI* were similar to $X_{f}^{i}$. Above all, we should focus on the occurrence and risk of Cd, Cu and Pb in Huang River and Cr in Yan River in the future.

## 4. Conclusions

The drinking and surface waters in five cities of northwestern China, Xi’an, Yan’an, Xining, Lanzhou and Urumqi, have been evaluated for their quality and risk based on MEs. A total of 161 water samples were collected from the residential taps and city rivers. All the samples were analyzed for 18 physicochemical water quality parameters. For pH, Cl^−^, SO_4_^2−^, Na^+^, K^+^, Ca^2+^, Mg^2+^, Cd, Cr and Cu, values were within the permissible limits, whereas F^−^, NO_3_^−^, NH_4_^+^ and Pb exceeded the permissible drinking water quality criteria of China. In terms of the surface water, F^−^, Cl^−^, NO_3_^−^, SO_4_^2−^ were above permissible limits according to the third range of the surface water quality suitability standard established for fishing and swimming in China. Thus, some water samples are not suitable for environmental health and this fact requires further study to find methods to correct the situation. These findings may help in developing targeted and sustainable cost-effective treatment technologies for remediation and water safety.

Water resources in the study area are similar and there are no clear variations for ions. In the case of DW, the difference for HMs may be caused by water pipes, faucets and their fittings. In general, the MEs except Cd and Cr are significantly higher in Xining drinking water than Xi’an. The Huang and Yellow rivers are affected by anthropogenic activities more seriously than the Yan River and Peaceful Canal. It’s worth noting that Huang resembles the Yellow River because Huang is one of the branches of the Yellow River.

The concentrations of Cd, Cr, Pb and Cu in drinking water influence health. The *HIt* of HMs decreases in the order of Cr > Cd > Pb > Cu in Xi’an and Xining, showing that the Cr levels were potentially deleterious to children health, mainly by the dermal exposure pathway. This is especially true in Xining where the 95% *HIt* value was even 0.98, which implies that the influence of toxic metals from tap water pipe systems on drinking water quality and safety should not be ignored. The carcinogenic risk from Cr exposure in Xi’an and Xining is at the permissible level. For the ecological assessment of the city’s rivers, comprehensive potential ecological risk index is in the order Xining > Urumqi > Yan’an > Lanzhou, and all ranked at low level of risk. It’s important to note that children’s health risks may be higher if more heavy metals are considered. In addition, this paper just studied the chemical indicators of one season, which would make more sense if subsequent seasonal variation and biological indicators was considered. Therefore, MEs and BOD variation associated with season, the bioaccessibility of MEs by microbiological influences, the integrated health risk due to MEs exposure to children, and epidemiologic effect caused from the EMs would be further studied in the future.

## Figures and Tables

**Table 1:** Parameters for HMs exposure in DW for children.

Item	Exposure Parameters	Value	Source
*C*	The concentration in DW	mg·L^−1^	In this study
*IngR*	Ingestion rate of water	0.327 L·day^−1^	[24]
*EF*	Exposure frequency	360 day·a^−1^	[25]
*ED*	Exposure duration	6 a^−1^	[24]
*BW*	Average body weight	15 kg	[24]
*AT*	Average exposure time	70 × 365 day	[24]
*SA*	Exposed skin area	1150 cm^2^	[26]
*AF*	Skin adherence factor	0.2 mg (cm^2^·day)^−1^	[24]
*ABS*	Dermal absorption factor	0.0003	[27]
*RfD*	The reference dose	mg·kg^−1^·day^−1^	[20]
*SF*	The slope factor	(mg/kg/day)^−1^	[20]

**Table 2:** Summary of *RfD* and *SF* of HMs.

Heavy Metals	Oral *RfD*	Dermal *RfD*	Oral *SF*	Dermal *SF*
Cd	0.0005	0.00001	NA	NA
Cr	0.003	0.003	0.501	20
Cu	0.04	0.012	NA	NA
Pb	0.0014	NF	NA	NA

Notes: NA—not applicable; NF—not found.

**Table 3:** Classification of $X_{f}^{i}$, $X_{d}$, $E_{r}^{i}$ and *RI*.

$X_{f}^{i}$	$X_{d}$	Contamination Levels	$E_{r}^{i}$	*RI*	Risk Levels
<1	<4	low	<30	<40	low
1–2	4–8	moderate	30–60	40–80	moderate
2–4	8–12	considerable	60–90	80–120	considerable
≥4	≥12	high	90–120	120–160	high
-	-	-	≥120	≥160	Very high

**Table 4:** An overview of MEs for DW and SW in the five major cities, NW, China.

Characteristics	Tap Water (*n* = 88)							Surface Water (*n* = 73)							Water Quality Criteria in China	
	Min．	25%	Median	75%	Max.	AV.	SD	Min.	25%	Median	75%	Max.	AV.	SD	Tap Water	Surface Water *
pH	7.1	7.8	8.0	8.1	8.5	7.9	0.2	7.0	7.5	7.8	8.0	9.0	7.8	0.4	6.5~8.5	6~9
EC (μS/cm)	114.0	126.3	135.5	291.0	428.0	204.0	92.1	209.0	316.5	417.0	979.0	3080.0	712.9	599.1		
F^−^ (mg/L)	0.0	0.1	0.1	0.3	3.1	0.4	0.6	0.1	0.3	0.7	1.2	2.9	0.8	0.6	1	1
Cl^−^ (mg/L)	4.6	8.2	9.4	14.3	38.3	12.0	6.2	12.5	35.1	81.6	151.4	604.8	125.7	122.8	250	250
HCO_3_^−^ (mg/L)	33.2	53.6	58.9	128.7	265.3	88.8	44.4	36.1	102.3	151.5	247.1	880.2	202.6	155.8		
NO^3−^ (mg/L)	5.0	9.7	11.2	15.8	26.4	13.0	4.3	1.4	13.4	17.9	37.3	315.0	39.9	67.0	10	10
SO_4_^2−^ (mg/L)	19.0	24.5	26.2	48.9	87.0	36.9	15.6	30.9	81.1	148.3	296.1	2172.9	274.4	335.2	250	250
NH_4_^+^ (mg/L)	0.0	0.0	0.2	0.5	1.8	0.3	0.4	0.0	0.0	0.0	0.0	3.0	0.1	0.4	0.5	1
K^+^ (mg/L)	1.1	1.5	1.9	2.5	9.3	2.3	1.4	0.2	2.5	5.5	12.1	97.5	11.6	16.2		
Na^+^ (mg/L)	2.1	2.9	4.1	11.3	115.7	8.4	12.9	8.1	33.1	78.3	175.2	1062.3	140.7	175.0	200	
Mg^2+^ (mg/L)	2.7	2.9	3.4	10.4	30.0	6.4	4.5	3.7	15.9	20.9	37.5	64.7	26.1	13.0		
Ca^2+^ (mg/L)	16.0	25.8	28.0	54.2	73.0	38.6	15.9	14.6	29.0	41.8	65.6	282.3	60.1	47.4		
Cd (μg/L)	0.0	0.1	0.1	0.2	3.6	0.2	0.4	0.0	0.0	0.1	0.1	0.3	0.1	0.1	5	5
Cr (μg/L)	0.2	1.6	2.5	3.2	7.3	2.5	1.2	0.4	2.1	3.3	6.2	31.7	4.8	4.7	50	50
Pb (μg/L)	0.0	0.4	1.1	2.2	14.7	1.6	2.1	0.0	0.3	0.5	1.4	49.0	2.7	8.1	10	50
Cu (μg/L)	0.6	3.5	5.6	8.8	161.4	10.7	24.0	0.2	2.5	5.1	9.0	51.6	7.2	7.6	1000	1000
TOC (mg/L)	1.6	2.9	3.5	3.9	151.6	5.5	16.1	0.6	2.6	3.5	6.1	47.3	5.2	6.3		
TN (mg/L)	4.2	5.8	6.7	8.0	15.8	7.3	2.0	1.1	8.1	13.1	22.5	100.0	21.5	21.9		

Note:* means the Third Range of Surface Water Quality Standard in China.

**Table 5:** Correlation Coefficient of MEs for DW in the five major cities, NW, China.

	pH	EC	F^−^	Cl^−^	HCO_3_^−^	NO_3_^−^	SO_4_^2−^	NH_4_^+^	K^+^	Na^+^	Mg^2+^	Ca^2+^	Cd	Cr	Pb	Cu	TOC
EC	−0.54 **																
F^−^	−0.12	0.44 **															
Cl^−^	−0.40 **	0.72 **	0.35 **														
HCO_3_^−^	−0.42 **	0.79 **	0.40 **	0.51 **													
NO_3_^−^	−0.29 **	0.71 **	0.32 **	0.72 **	0.53 **												
SO_4_^2−^	−0.44 **	0.88 **	0.47 **	0.84 **	0.75 **	0.83 **											
NH_4_^+^	0.13	0.01	−0.18	0.02	−0.05	0.33 **	0.09										
K^+^	−0.40 **	0.72 **	0.38 **	0.69 **	0.74 **	0.52 **	0.76 **	0.04									
Na^+^	−0.37 **	0.85 **	0.45 **	0.80 **	0.81 **	0.72 **	0.92 **	0.07	0.86 **								
Mg^2+^	−0.36 **	0.81 **	0.43 **	0.63 **	0.85 **	0.66 **	0.84 **	0.00	0.75 **	0.87 **							
Ca^2+^	−0.49 **	0.80 **	0.39 **	0.52 **	0.98 **	0.56 **	0.76 **	−0.07	0.72 **	0.78 **	0.83 **						
Cd	0.06	−0.21	−0.33 **	−0.11	−0.26 *	−0.08	−0.16	0.30 **	0.03	−0.12	−0.23 *	−0.24 *					
Cr	0.08	−0.36 **	−0.16	−0.30 **	−0.41 **	−0.41 **	−0.38 **	−0.07	−0.22*	−0.36 **	−0.42 **	−0.40 **	0.37 **				
Pb	−0.25 *	0.63 **	0.15	0.49 **	0.55 **	0.44 **	0.59 **	−0.16	0.50 **	0.65 **	0.61 **	0.54 **	−0.23	−0.40 **			
Cu	−0.14	0.15	0.04	0.09	0.28 **	0.20	0.20	−0.09	0.28 **	0.20	0.15	0.32 **	0.26 *	−0.13	0.13		
TOC	0.21 *	−0.41 **	−0.36 **	−0.20	−0.36 **	−0.27 *	−0.33 **	0.17	−0.17	−0.31 **	−0.45 **	−0.36 **	0.52 **	0.36 **	−0.37 **	0.13	
TN	−0.49 **	0.75 **	0.32 **	0.69 **	0.62 **	0.74 **	0.82 **	0.13	0.67 **	0.78 **	0.70 **	0.66 **	0.06	−0.28 **	0.50 **	0.30 **	−0.23 *

Notes: * Correlation is significant at the 0.05 level (2-tailed). ** Correlation is significant at the 0.01 level (2-tailed).

**Table 6:** Correlation Coefficient of MEs for SW in the five major cities, NW, China.

	pH	EC	F^−^	Cl^−^	HCO_3_^−^	NO_3_^−^	SO_4_^2−^	NH_4_^+^	K^+^	Na^+^	Mg^2+^	Ca^2+^	Cd	Cr	Pb	Cu	TOC
EC	−0.56 **																
F^−^	0.12	−0.13															
Cl^−^	−0.35 **	0.56 **	0.09														
HCO_3_^−^	−0.20	0.34 **	0.01	0.67 **													
NO_3_^−^	0.02	−0.05	0.15	0.22	0.18												
SO_4_^2−^	−0.26 *	0.51 **	0.07	0.96 **	0.72 **	0.17											
NH_4_^+^	−0.02	0.18	−0.40 **	−0.13	−0.15	−0.18	−0.14										
K^+^	−0.34 **	0.45 **	0.03	0.67 **	0.55 **	0.01	0.65 **	−0.03									
Na^+^	−0.27 *	0.40 **	0.12	0.88 **	0.68 **	0.21	0.85 **	−0.13	0.74 **								
Mg^2+^	−0.13	0.07	0.10	0.68 **	0.52 **	0.32 **	0.69 **	−0.32 **	0.55 **	0.74 **							
Ca^2+^	−0.39 **	0.54 **	−0.22	0.59 **	0.55 **	0.14	0.54 **	0.02	0.58 **	0.52 **	0.42 **						
Cd	−0.09	0.33 **	−0.24	0.02	0.10	−0.32 *	0.05	0.24	0.38 **	0.01	−0.22	0.30 *					
Cr	0.09	−0.07	−0.07	0.14	0.06	−0.03	0.12	0.08	0.29 *	0.17	0.25 *	0.20	−0.05				
Pb	0.13	0.04	0.22	0.13	0.33 *	−0.13	0.15	0.00	0.35 **	0.09	0.09	0.06	0.33 *	0.04			
Cu	0.08	0.13	−0.09	0.38 **	0.39 **	−0.28 *	0.41 **	0.07	0.61 **	0.38 **	0.28 *	0.34 **	0.34 **	0.39 **	0.41 **		
TOC	−0.30 *	0.40 **	0.08	0.56 **	0.57 **	−0.18	0.58 **	−0.06	0.74 **	0.58 **	0.30 *	0.53 **	0.47 **	0.06	0.35 **	0.57 **	
TN	−0.40 **	0.35 **	0.06	0.69 **	0.47 **	0.41 **	0.60 **	−0.24	0.68 **	0.74 **	0.64 **	0.58 **	0.07	0.14	0.07	0.28 *	0.53 **

Notes: * Correlation is significant at the 0.05 level (2-tailed). ** Correlation is significant at the 0.01 level (2-tailed).

**Table 7:** Summary of Xi’an (*n* = 50) non-carcinogenic Hazard Index (*HI*) and carcinogenic Incremental Lifetime Cancer Risk (*ILCR*) via ingestion and dermal exposure of DW based on HMs at the 5th, median and 95th percentiles.

**Heavy Metals Noncarcinogenic Effects (*HI*)**	**5%**			**Median**			**95%**		
	***HIing***	***HIder***	***HIt***	***HIing***	***HIder***	***HIt***	***HIing***	***HIder***	***HIt***
Cd	2.26 × 10^−4^	2.38 × 10^−3^	2.61 × 10^−3^	4.32 × 10^−4^	4.56 × 10^−3^	4.99 × 10^−3^	1.81 × 10^−3^	1.91 × 10^−2^	2.09 × 10^−2^
Cr	1.13 × 10^−3^	3.75 × 10^−1^	3.76 × 10^−1^	1.74 × 10^−3^	5.79 × 10^−1^	5.81 × 10^−1^	2.60 × 10^−3^	8.67 × 10^−1^	8.70 × 10^−1^
Cu	4.82 × 10^−5^	3.39 × 10^−5^	8.22 × 10^−5^	2.24 × 10^−4^	1.58 × 10^−4^	3.82 × 10^−4^	1.70 × 10^−3^	1.19 × 10^−3^	2.89 × 10^−3^
Pb	-	-	-	7.18 × 10^−5^	1.52 × 10^−5^	8.70 × 10^−5^	3.54 × 10^−3^	7.46 × 10^−4^	4.28 × 10^−3^
Total	1.55 × 10^−3^	3.85 × 10^−1^	3.87 × 10^−1^	2.73 × 10^−3^	5.89 × 10^−1^	5.92 × 10^−1^	9.95 × 10^−3^	8.77 × 10^−1^	8.82 × 10^−1^
**Heavy Metals Carcinogenic Risks (*ILCR*)**	**5%**			**Median**			**95%**		
	***HIing***	***HIder***	***sum***	***HIing***	***HIder***	***sum***	***HIing***	***HIder***	***sum***
Cr	1.69 × 10^−6^	1.43 × 10^−5^	1.59 × 10^−5^	2.61 × 10^−6^	2.20 × 10^−5^	2.46 × 10^−5^	3.91 × 10^−6^	3.29 × 10^−5^	3.69 × 10^−5^

Note: - means the values are below the detection limit.

**Table 8:** Summary of Xining (*n* = 38) non-carcinogenic Hazard Index (*HI*) and carcinogenic Incremental Lifetime Cancer Risk (*ILCR*) via ingestion and dermal exposure of DW based on HMs at the 5th, median and 95th percentiles.

**Heavy Metals Noncarcinogenic effects (*HI*)**	**5%**			**Median%**			**95%**		
	***HIing***	***HIder***	***HIt***	***HIing***	***HIder***	***HIt***	***HIing***	***HIder***	***HIt***
Cd	8.77 × 10^−5^	9.26 × 10^−4^	1.01 × 10^−3^	3.02 × 10^−4^	3.19 × 10^−3^	3.49 × 10^−3^	1.04 × 10^−3^	1.09 × 10^−2^	1.20 × 10^−2^
Cr	3.45 × 10^−4^	1.15 × 10^−1^	1.15 × 10^−1^	9.26 × 10^−4^	3.09 × 10^−1^	3.10 × 10^−1^	2.93 × 10^−3^	9.77 × 10^−1^	9.80 × 10^−1^
Cu	6.30 × 10^−5^	4.43 × 10^−5^	1.07 × 10−^4^	3.04 × 10^−4^	2.14 × 10^−4^	5.17 × 10^−4^	1.34 × 10^−3^	9.46 × 10^−4^	2.29 × 10^−3^
Pb	2.22 × 10^−4^	4.69 × 10^−5^	2.69 × 10^−4^	2.06 × 10^−3^	4.34 × 10^−4^	2.49 × 10^−3^	5.91 × 10^−3^	1.25 × 10^−3^	7.15 × 10^−3^
Total	2.13 × 10^−3^	1.16 × 10^−1^	1.18 × 10^−1^	4.46 × 10^−3^	3.16 × 10^−1^	3.22 × 10^−1^	1.09 × 10^−2^	9.82 × 10^−1^	9.88 × 10^−1^
**Heavy Metals carcinogenic risks (*ILCR*)**	**5%**			**Median**			**95%**		
	***HIing***	***HIder***	***sum***	***HIing***	***HIder***	***sum***	***HIing***	***HIder***	***sum***
Cr	5.18 × 10^−7^	4.36 × 10^−6^	4.88 × 10^−6^	1.39 × 10^−6^	1.17 × 10^−5^	1.31 × 10^−5^	4.41 × 10^−6^	3.71 × 10^−5^	4.15 × 10^−5^

## Supplementary Materials

The following are available online at www.mdpi.com/1660-4601/14/10/1168/s1, Figure S1: Spatial distributions of cations, anions and HMs of DW and SW in Xi’an, Yan’an, Lanzhou and Urumqi, Figure S2: Boxplots comparing MEs of DW and SW in the study areas, Figure S3: Spatial distributions of total hazard index (*HIt*) of DW in Xi’an (a) and Xining (b).
