# Supplementary material for: Multi-Elements in Source Water (Drinking and Surface Water) within Five Cities from the Semi-Arid and Arid Region, NW China: Occurrence, Spatial Distribution and Risk Assessment
Source: Int J Environ Res Public Health. 2017 Oct 2;14(10):1168. doi: 10.3390/ijerph14101168 (PMC5664669; doi:10.3390/ijerph14101168)
Supplement: Supplementary file 1 [file ijerph-14-01168-s001.pdf]

International Journal of
Environmental Research and Public Health

MDPI

Supplementary Materials: Multi-elements in Source Water (Drinking and Surface Water) within Five Cities from the Semi-Arid and Arid Region, NW China: Occurrence, Spatial Distribution and Risk Assessment

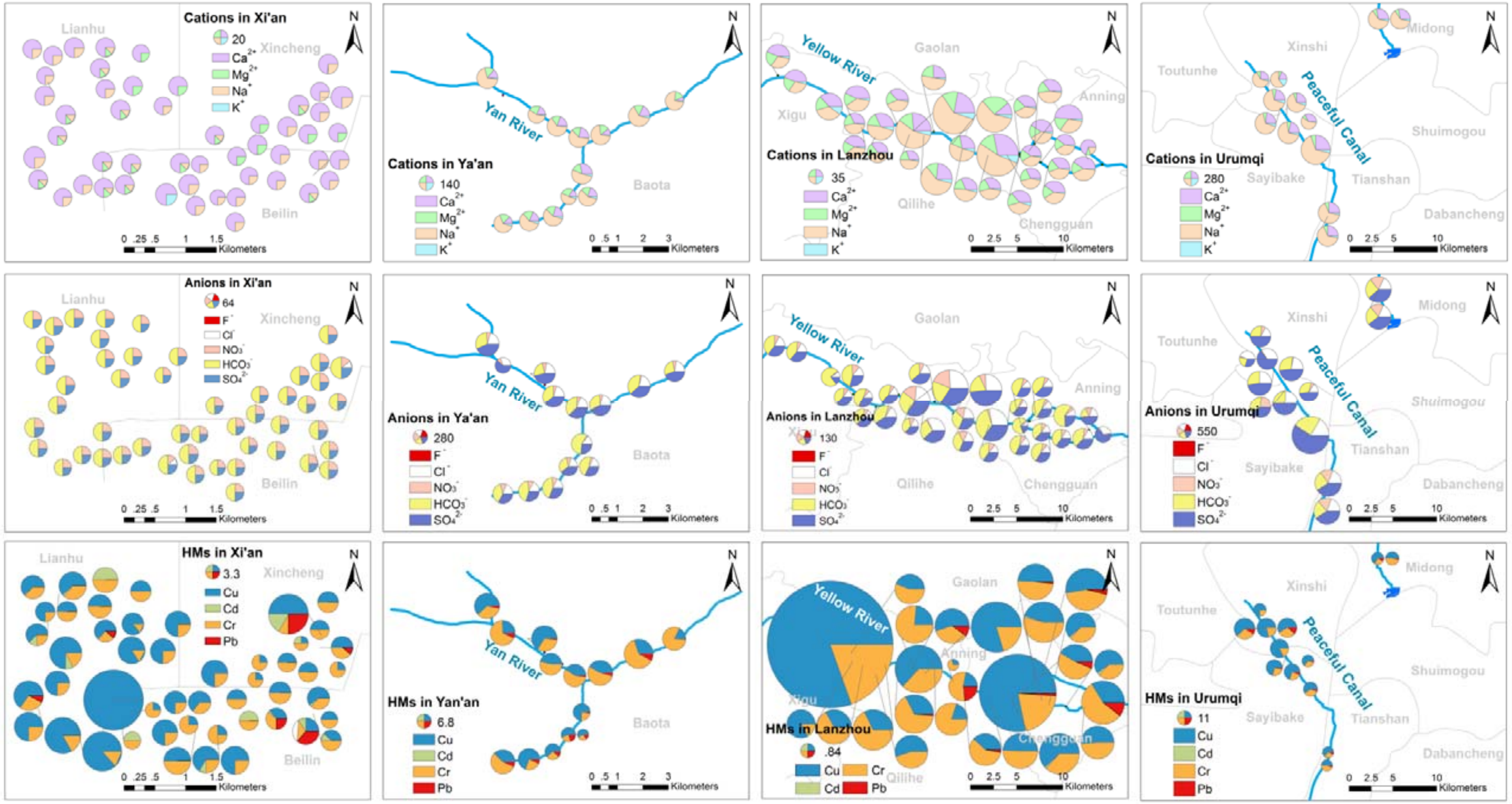

Figure S1. Spatial distributions of cations, anions and HMs of DW and SW in Xi'an, Yan'an, Lanzhou and Urumqi.

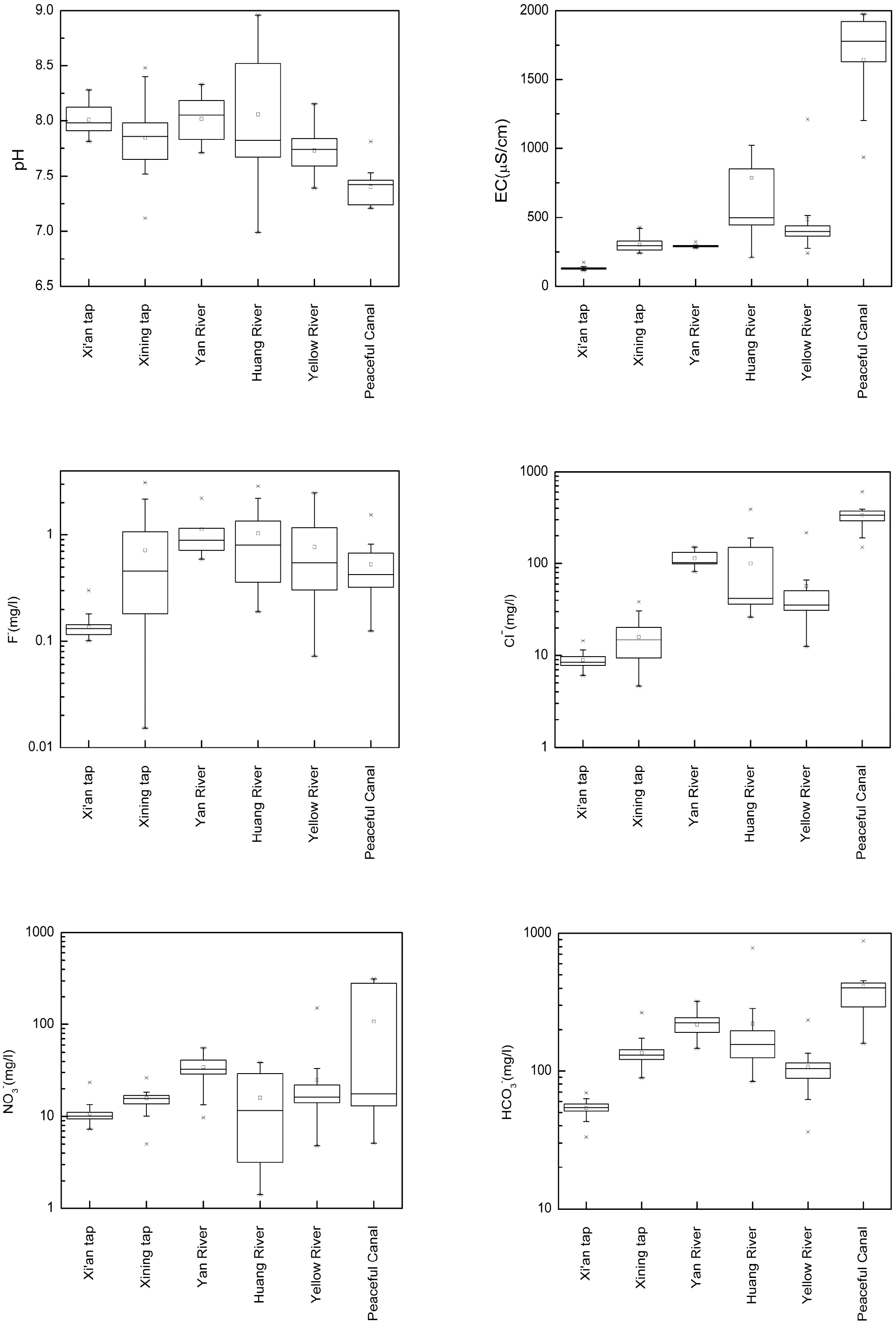

Figure S2. *Cont.*

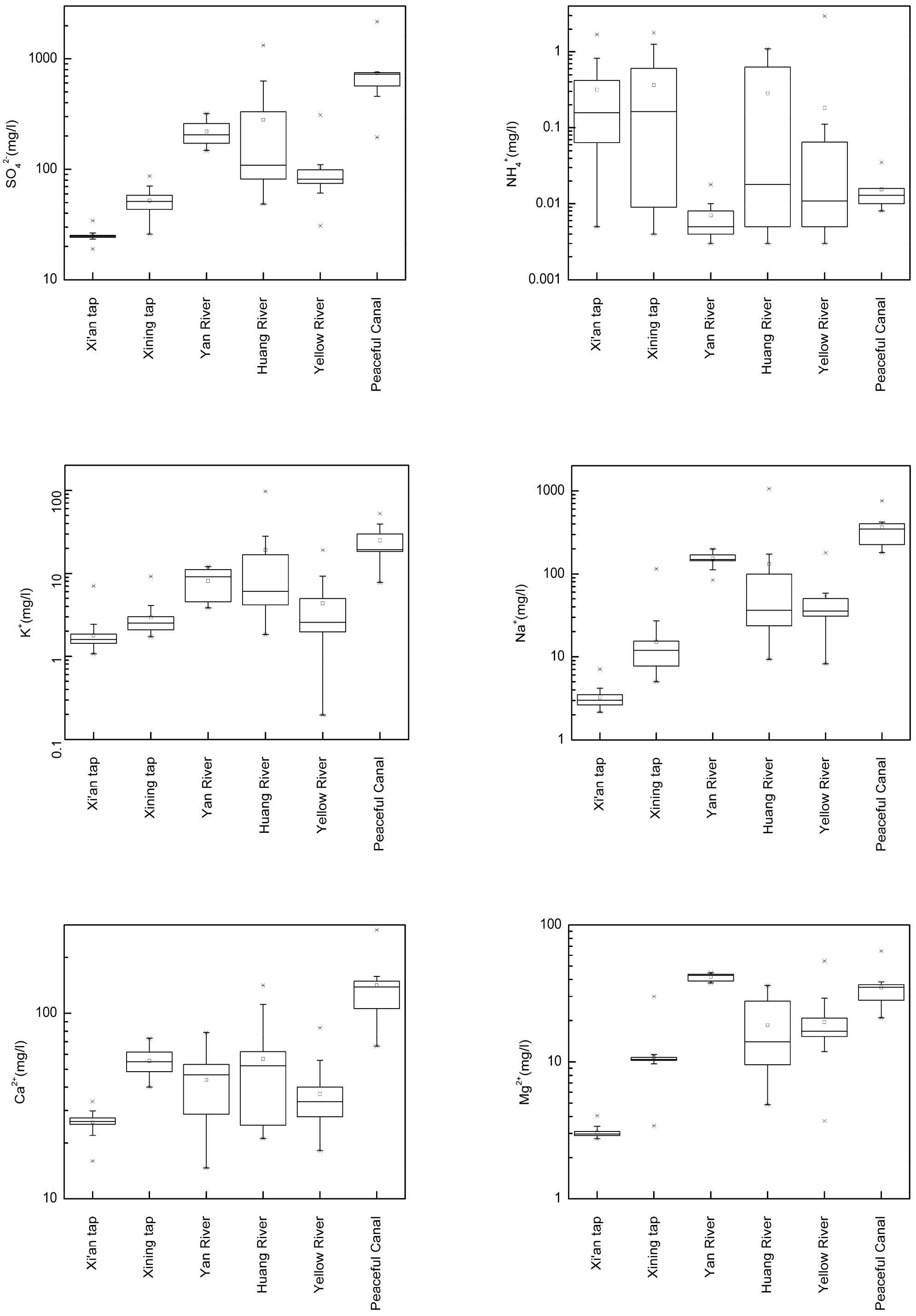

Figure S2. *Cont.*

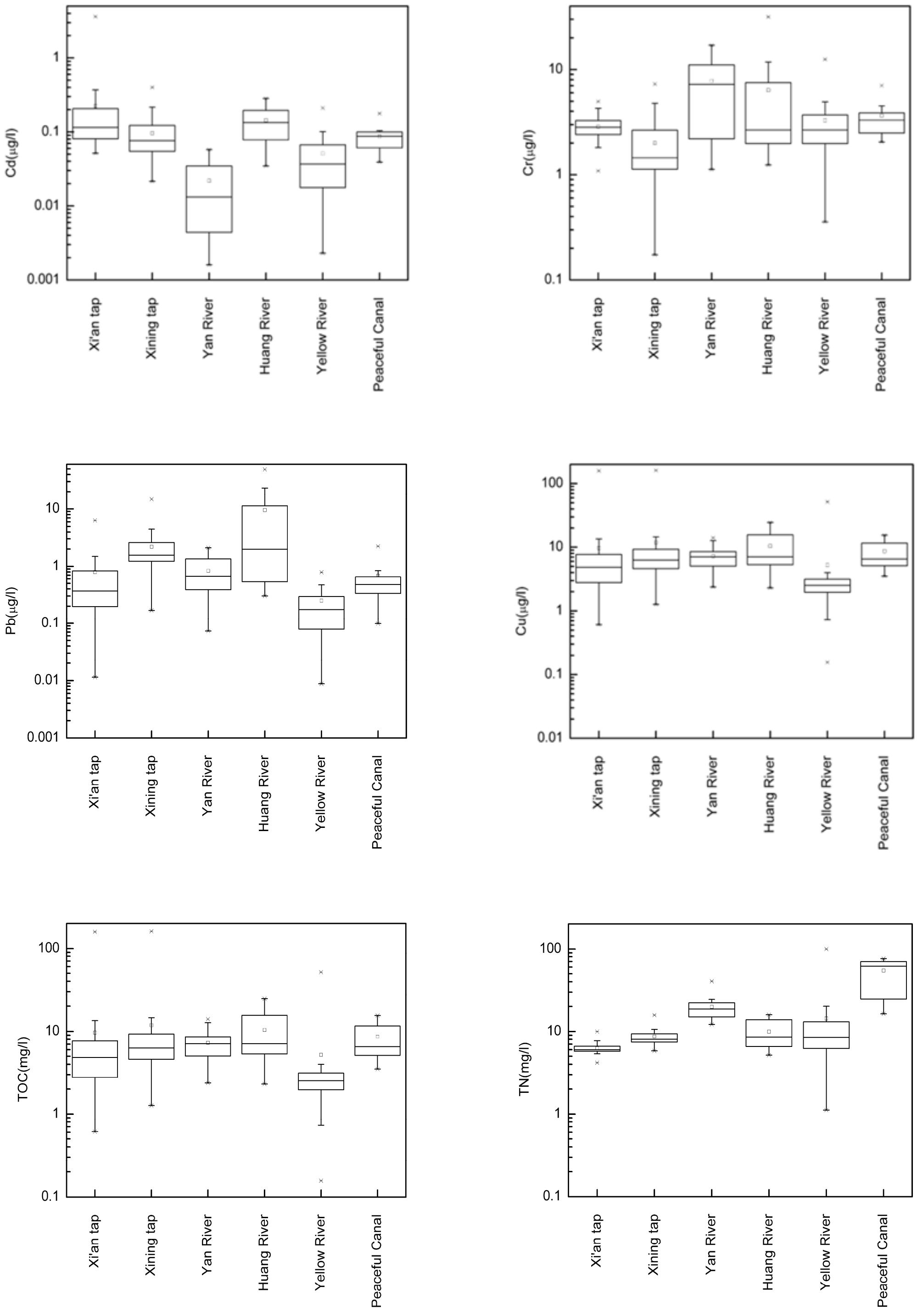

Figure S2. Boxplots comparing MEs of DW and SW in the study areas.

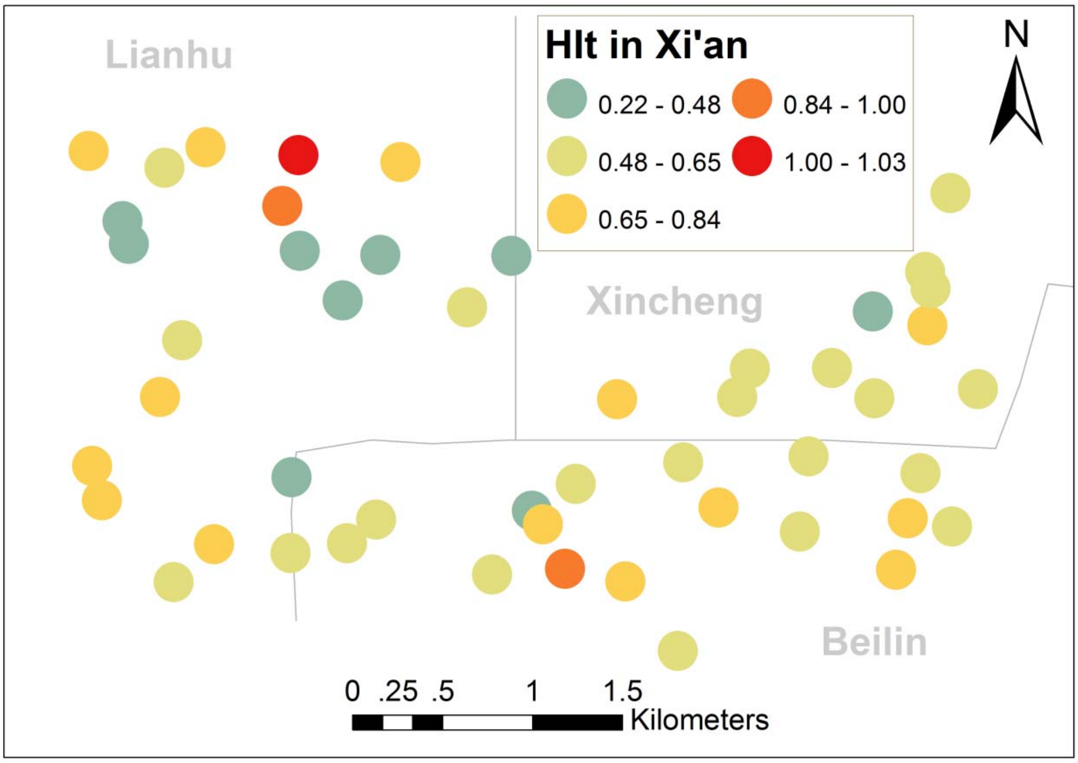

(**a**)

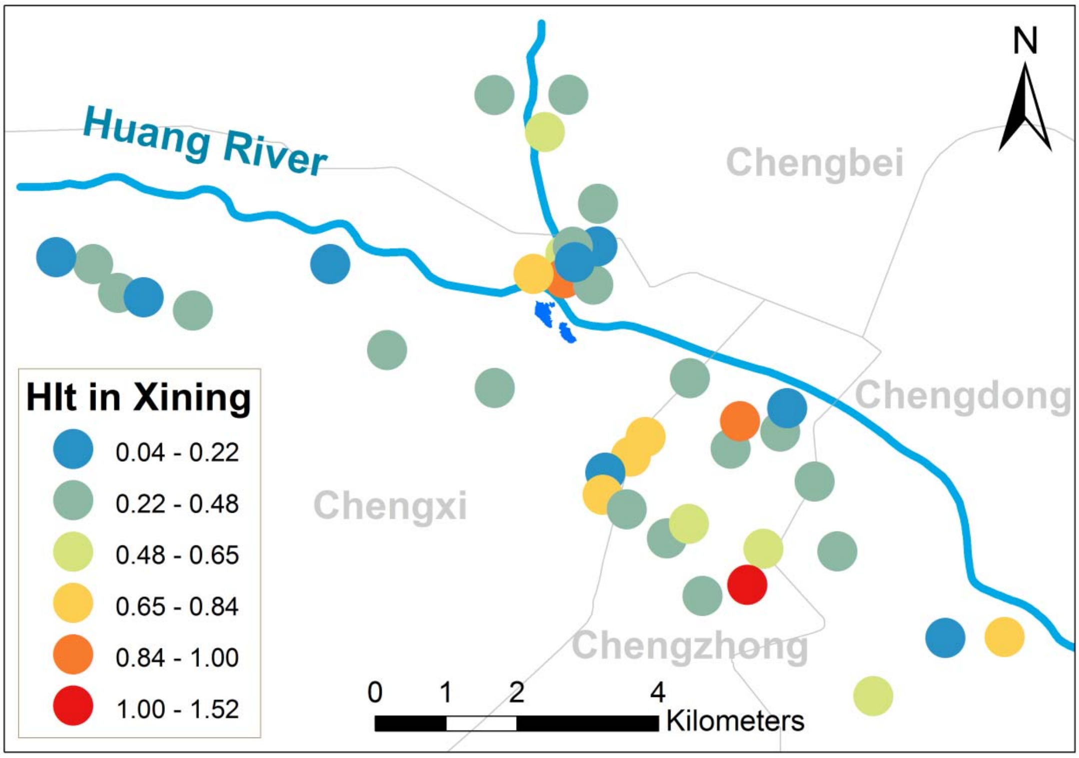

(**b**)

Figure S3. Spatial distributions of total hazard index (HIt) of DW in Xi'an (**a**) and Xining (**b**).
